# Development of an Educational Brochure about Treatment Options for Pregnant Women with Opioid Use Disorders

**DOI:** 10.3390/pharmacy12040097

**Published:** 2024-06-22

**Authors:** Ruth Jeminiwa, Sohyeon Park, Caroline Popielaski, Meghan Gannon, Ronald Myers, Diane J. Abatemarco

**Affiliations:** 1 Jefferson College of Pharmacy, Thomas Jefferson University, Philadelphia, PA 19107, USA; sohyeon.park@students.jefferson.edu (S.P.); caroline.popielaski@students.jefferson.edu (C.P.); 2 Department of Obstetrics and Gynecology, Thomas Jefferson University, Philadelphia, PA 19107, USA; meghan.gannon@jefferson.edu (M.G.); dja007@jefferson.edu (D.J.A.); 3 Department of Medical Oncology, Thomas Jefferson University, Philadelphia, PA 19107, USA; ronald.myers@jefferson.edu

**Keywords:** patient education, opioid use disorders, pregnancy, treatment options

## Abstract

The goal of this study was to describe the development of an educational brochure for pregnant women with opioid use disorders (OUDs) about treatment options. Based on findings from a preliminary review of the literature, we drafted a brochure that addressed the following questions: (1) What are your options (Medication-Assisted Treatment (MAT) versus no treatment)? (2) What are the benefits of MAT? (3) What are the risks of MAT? (4) Can I take buprenorphine or methadone while breastfeeding? (5) Which medication should I choose? Clinicians and doulas (n = 19) who provide care to pregnant women with OUDs were recruited. Semi-structured interviews elicited participants’ feedback on brochure content and their perceptions about brochure use for patient education. Thematic data analyses were performed. Three emergent themes were identified (suggested uses and settings of use, content revisions, and perceptions about the brochure) and used to refine the final brochure. This study provides valuable insights into the desired content of an educational brochure describing treatment options for pregnant women with OUDs from the provider’s standpoint. Research is needed to assess the use of the brochure in shared decision-making conversations with providers about treatment.

## 1. Introduction

Rising cases of opioid use disorder (OUD) in pregnant women in the United States are leading to increased rates of neonatal abstinence syndrome (NAS) and maternal opioid-related diagnoses, about 82.5% and 134%, respectively, from 2010 to 2017 [1]. The increased incidence of these two conditions may lead to higher healthcare costs. As of 2012, NAS alone led to hospital cost expenditure of USD 1.5 billion [2]. Considering that pregnant women diagnosed with OUDs are at greater risk for preterm labor, stillbirth, and maternal death [3], additional considerations regarding treatment and care may help [4]. Though there has been an increase in treatment initiatives and preventative efforts and a rising trend in the utilization of medication-assisted treatment (MAT), gaps in care and treatment for this special population still persist [1,5]. In 2015, only 56% of pregnant women with OUDs on Medicaid (a government program that provides insurance coverage to low-income individuals in the US) received MAT in Pennsylvania [5].

As these women engage with healthcare providers during their pregnancies, there are opportunities to address some of the factors that may impact access to appropriate care and MAT use. The Substance Abuse and Mental Health Services Administration (SAMHSA) and the American College of Obstetricians and Gynecologists guidelines recommend patient education for the treatment of pregnant women with OUDs [6,7]; clinicians are urged to discuss the risks and benefits of treatment before arriving at a mutually agreed treatment path. As a result, there have been various patient education interventions developed for this special population. One study developed a NAS education program that targeted breastfeeding rates [8]. Another study created an integrated program that involved the distribution of patient education materials to community partners, as well as the patients [9]. For community partners, referral workflow guides were given to improve the referral process and reduce errors. For the patients, a program information card was used to advertise this clinic’s integrated program and provide general harm reduction tips to promote a positive attitude towards treatment during pregnancy and reducing use [9]. While helpful, these tools do not provide sufficient information women need to make well-informed decisions about treatment.

Pregnant women with OUDs often lack information or receive misinformation about OUD treatment and its impact on themselves and their babies [10]. A lack of adequate communication, breakdown in communication, or miscommunication experienced by pregnant women with opioid use disorders worsens perceptions of stigma from clinicians [11]. Therefore, the intentional education of patients about treatment options may reduce misinformation and stigma and may facilitate shared decision making.

Pregnant women with opioid use disorders desire information from trusted sources about treatments and their impact [10]. Furthermore, evidence-based guidelines like the American Society of Addiction Medicine’s guideline for treating this patient population recommend shared decision making, which requires patient education about the risks and benefits of treatment options [12]. Therefore, this research sought to bridge the gap between the lack of trusted, evidence-based, and accessible information about the risks and benefits of treatment options for pregnant women with opioid use disorders.

Although existing materials have considered the risks of opioid use in pregnancy, the use of methadone to treat opioid dependence, and common facts about treatment for opioid use disorders, evidence-based resources that provide information about the risks and benefits of treatment versus non-treatment for pregnant women with OUDs are scarce [13,14,15]. The primary objective of this research was to develop an evidence-based brochure to educate pregnant women about treatment options at points of care to facilitate patient autonomy, agency, and shared decision making. A brochure was chosen because patient information leaflets are associated with improved patient knowledge and satisfaction [16]. A secondary objective was to elicit clinicians’ opinions on the content and use of the brochure in clinical practice.

## 2. Materials and Methods

The research team included doctoral-level researchers and professional PharmD students with training in qualitative research. RJ, MG, and DA study the treatment of pregnant women with OUDs, while another member of the team (RM) is a shared decision-making expert.

### 2.1. Brochure Development

One of the researchers, RJ, performed a preliminary review of peer-reviewed articles via Google Scholar and PubMed to inform a brochure draft outlining the benefits and risks of receiving MAT in pregnancy versus not receiving it [17,18,19]. The Consolidated Criteria for Reporting Qualitative Research (COREQ) was followed in the conduct and reporting of this study [20].

**Figure A1:**
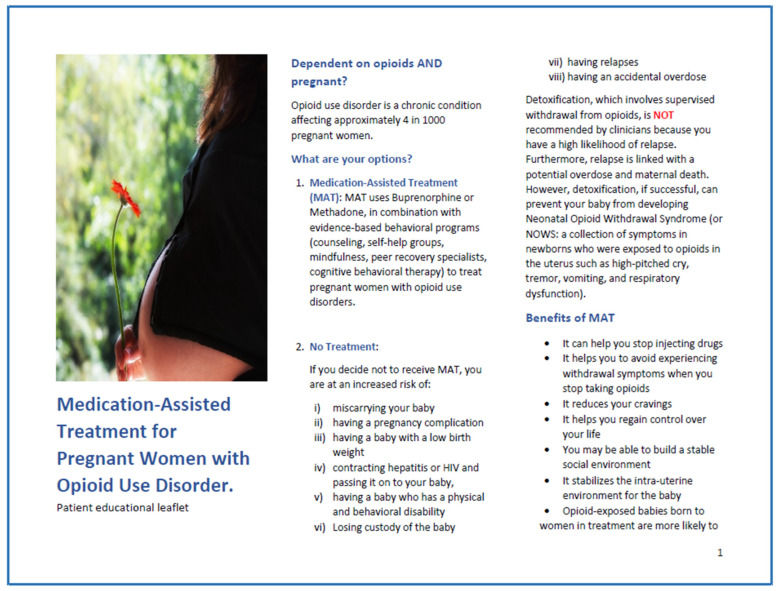

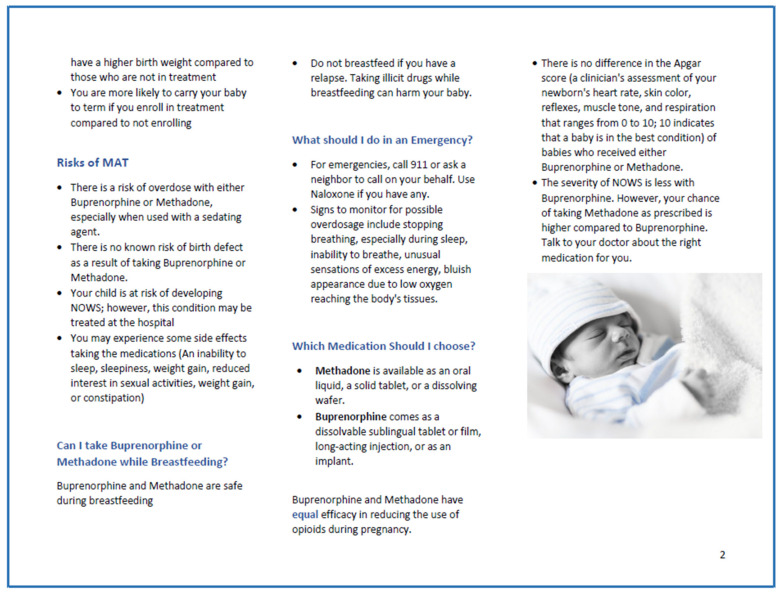
The first version of a tri-folding brochure.

After reviewing the literature, we drafted a brochure (Appendix A, Figure A1) containing the following information: (1) What are your options (MAT versus no treatment)? (2) Benefits of MAT. (3) Risks of MAT. (4) Can I take buprenorphine or methadone while breastfeeding? (5) Which medication should I choose? The team then developed a plan to present the brochure to resident and attending physicians in family medicine and obstetrics and gynecology as well as professional doulas to evaluate its practicality and to gather their valuable input on its content and perceptions of use in practice. The study was part of a larger shared decision-making study.

### 2.2. Participant Recruitment

Attending physicians or residents and professional doulas who provide care to pregnant women with OUDs were recruited purposively. Emails with information about the study were sent to family medicine, obstetrics, and gynecology departments of two academic medical centers and a doula network service in the northeastern part of the United States of America. The email lists were obtained from contacts from the respective programs. Interested participants contacted the primary investigator. The study was approved by our institution’s review board. Participants provided verbal and signed informed consent and were given a USD 100 gift card to compensate them for their time. After providing informed consent, participants were directed to complete an online survey, eliciting their sociodemographic characteristics. One person declined participation due to a busy schedule.

### 2.3. Semi-Structured Interviews

Semi-structured interviews elicited participants’ feedback on the brochure’s content and their perceptions about using it for patient education. The first author (R.J.), a female pharmacy educator with a doctoral degree in pharmaceutical sciences, health outcomes research, and policy options, conducted the interviews. There was no prior relationship or familiarity between the interviewer and participants. The interviews were conducted virtually using the Zoom teleconferencing application, with only the interviewer and each participant present. Interviews were audio recorded and transcribed verbatim using a transcription service. Feedback on the brochure’s content was elicited as part of interviews for a separate study assessing the barriers and facilitators for using shared decision-making to support treatment decisions for pregnant women with opioid use disorders. Participants were asked to look at the brochure and provide their thoughts on using it to educate patients. Participants also read the brochure and provided feedback on its content. The length of the interviews was 1 h on average.

### 2.4. Data Analyses

Participants’ sociodemographic characteristics were described using descriptive statistics. Qualitative data were analyzed using NVIVO 12 software (QSR International Pty Ltd. Burlington, Massachusetts, USA. 2020). Two authors, R.J. and C.P., reviewed the transcripts repeatedly to become familiar with the data. Thematic analyses were performed. R.J. first noted initial codes while reviewing the transcripts. C.P. also reviewed the transcripts and either revised or added new codes to create a codebook. The final codes were achieved after review by the authors. R.J. and C.P. coded the transcripts independently using the code book to ensure reliability. Each author utilized NVIVO 12 software in coding. Both authors met to compare their coding. Discrepancies were resolved by consensus. R.J. reviewed the codes and categorized them into themes and subthemes based on their conceptual similarities. The final themes were arrived at after discussing emergent themes and subthemes with C.P., and with agreement from other research team members.

### 2.5. Brochure Revision

**Figure A2:**
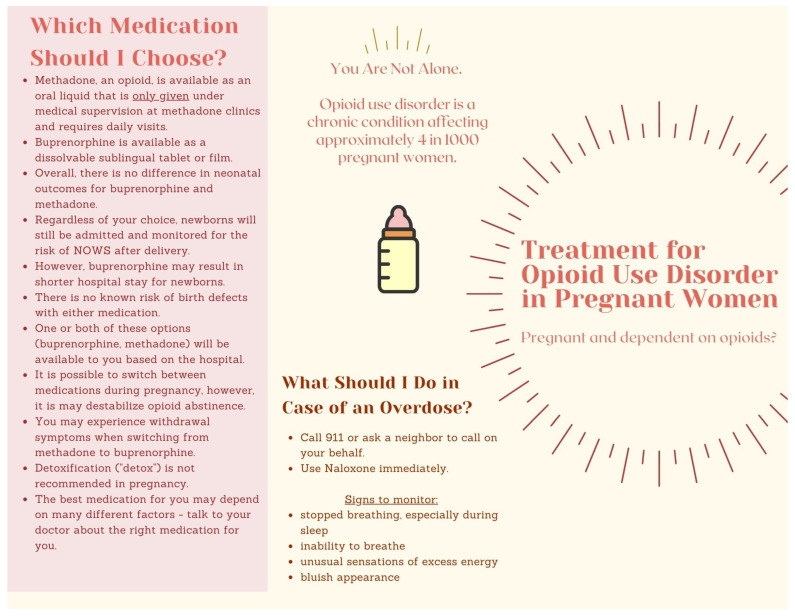

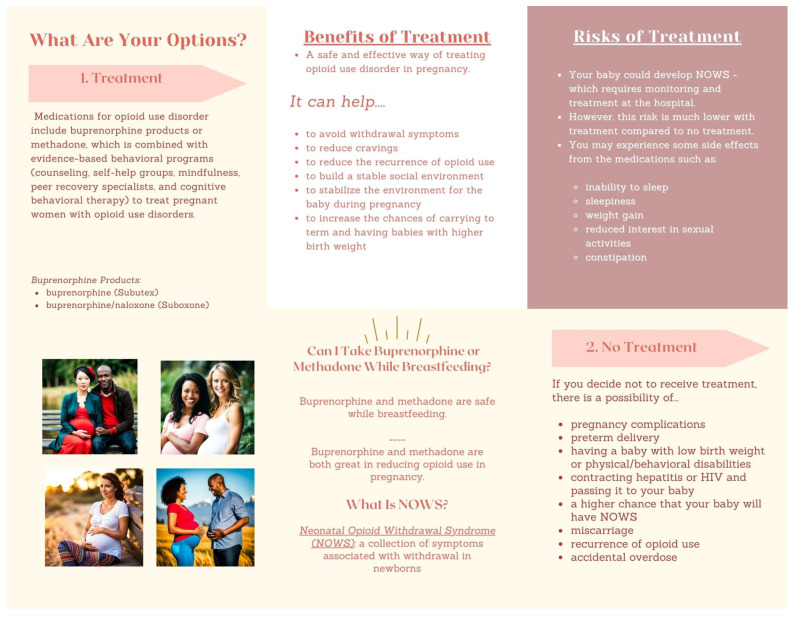
Revised version of a tri-folding brochure.

S.P., a co-author, implemented suggested changes from research participants in the brochure to create the updated version (Appendix A, Figure A2). R.J., another author, reviewed the final draft to ensure that the recommended changes were made.

## 3. Results

A total of nineteen participants were interviewed. Our participants were 35 years old on average and mostly female. They included six doulas, seven residents, and six attending physicians. The characteristics of the qualitative study participants are described in Table A1. There were three emergent themes from the analysis: (1) suggested uses and settings of use, (2) content revisions, and (3) perceptions about the brochure. Please see Appendix B, Table A2 for the themes, subthemes, and participant quotes.

### 3.1. Themes

#### 3.1.1. Suggested Uses and Settings of Use

This theme had two subthemes: settings of use and patient education.

- Settings of use: Several participants thought the brochure could be used in the emergency room, outpatient clinics, and community spaces, such as train stations, bus stations, churches, and needle exchange locations. One person noted that the brochure may be needed in other languages. Participants believed that although the prenatal clinic was a suitable place to have the brochure, most patients may not visit the outpatient clinic as new patients. Some participants thought the brochure was great for undecided patients, those not yet ready to engage in treatment, and those contemplating treatment but had not yet committed to anything.
- Brochure uses: Some participants also felt that the brochure could be used to educate patients about options for treatment or facilitate patient counseling. The brochure could also serve as take-home information after counseling or for individuals who are not somnolent. However, in general, participants felt the brochure should not be used as a handout without education or counseling on its content with patients. Patients could refer to the brochure to recollect information shared with them during the visit.

#### 3.1.2. Content Revisions

Four subthemes were identified for this theme: (a) suggestions to provide additional information, (b) perceptions on including information on detoxification, (c) wording of content, and (d) pictures.

- Suggestions to provide additional information

Participants suggested additional information for inclusion in the brochure.

(1) Additional information about Medications for Opioid Use Disorder: There were suggestions to include information about daily visits for methadone and the fact that buprenorphine could be prescribed for longer periods. One person mentioned that patients do not often have this information and are surprised when they are asked to visit the clinic daily to receive methadone. Several participants also mentioned that useful information would include the specific eligibility criteria for prescribing buprenorphine to patients. The eligibility criteria are dependent on patient treatment histories and specific organization criteria. For example, having an incarceration record disqualified people from receiving buprenorphine in one of the hospitals where a participant works. Many participants pointed out the need to include the brand names for buprenorphine products (Subutex and Suboxone) in parenthesis since that was how patients identified those products. In the draft, all dosage forms for each medication were listed. Some participants mentioned the need to specify the dosage form for each medication that is currently available for patients instead of dosage forms that have not been used or tested in pregnant women with OUDs. For example, even though methadone is available as a tablet, oral solution, injection, and powder, only the oral solution is offered to patients.
(2) Contact information: Participants thought that good contact information about the institution providing the brochure was essential and could be referred to by the patient. Additionally, good contact information specifies an affiliation, which could increase the patient’s trust in the brochure’s content.
(3) Prioritizing pregnant women: A participant suggested including a statement about pregnancy being the best time to receive treatment, especially because treatment programs often prioritize pregnant women.
(4) Breastfeeding: Another suggestion pertained to including additional information about breastfeeding. One participant mentioned that it should be included that breastfeeding while receiving treatment could help treat withdrawal in babies. This was more so in cases where there were no contraindications. Overall, several participants thought that the safety and benefits of breastfeeding while receiving treatment should be emphasized.
(5) APGAR score: The draft brochure contained information about APGAR to depict the baby’s health outcomes. Some people believed the word APGAR should be removed, while others felt there should be a definition of the term for ease of understanding.
(6) Neonatal opioid withdrawal syndrome (NOWS): The brochure used the acronym NOWS to depict neonatal opioid withdrawal syndrome. A few participants suggested that the acronym should be written out in full. Some people thought it should be emphasized that the risk for NOWS was lower with treatment compared to no treatment.

- Perceptions on including information on detoxification

There were several suggestions regarding detoxification. Many people did not support including detoxification as an option since it is not recommended for pregnant women by the current clinical practice guidelines. However, some noted that patients know about detoxification as a treatment option and often ask them about it. Therefore, they argued that the brochure should include detoxification as a treatment option. One person mentioned that detoxification should not be presented as though it were not an option for the patient. Rather, the brochure should help the patients arrive at that conclusion themselves. There was also a suggestion to put the word detox in parenthesis since it was a more popular term than detoxification.

- Wording of Content
  (1) Risk of treatment: The most common feedback received from participants was recommendations to reword or reframe information about the risks of treatment. Several participants suggested that the risks associated with no treatment should be written differently by rewording or changing the order. Participants felt the description of the risks of no treatment sounded punitive and scary and may turn some people away. Some participants thought the information in the brochure should be reframed to emphasize the benefits of receiving treatment instead of the risks of no treatment. In general, participants wanted the safety and benefits of treatment, especially medications for OUDs, to be emphasized.
  (2) Reading level: There were suggestions to reword some content to ensure the reading level was appropriate for sixth-grade readers.
- Pictures

Several participants believed the picture of the pregnant woman in the brochure should be changed. The reasons for this opinion were different. A few people felt that the woman in the picture was white, and that excluded people with a different skin tone. Rather, it was suggested that a silhouette cartoon or picture without skin tone with a pregnancy should be used. Someone pointed out that most women with opioid use disorders do not have a well-advanced pregnancy, so a less advanced pregnancy picture should be used. The draft brochure had a picture of a baby, which one participant thought should be changed since the baby’s skin tone looked like the baby was not alive. Apart from suggestions to change pictures, there were also suggestions to reduce the textual information but increase the pictures to make the brochure more appealing to patients. Patients could then be directed to a website with additional information via a link on the brochure.

#### 3.1.3. Perceptions about the Brochure

Participants expressed positive feedback about the brochure. Some people thought the brochure was easy to read. Several participants believed the brochure contained pertinent information needed to address patient concerns or to educate patients about treatment options.

The brochure was revised based on feedback from physicians and professional doulas. We changed the picture of the pregnant woman to capture diversity with images of women with diverse ethnicities. We also replaced the picture of a baby with a feeding bottle which suggests a baby, without showing one. The word “Medication Assisted Treatment” was replaced as suggested by a participant. There were several changes to more appropriately capture the content recommended by clinicians and doulas. We also modified the color and design of the brochure to better appeal to women.

## 4. Discussion

This paper describes the development of an educational brochure that describes treatment options for pregnant women with OUDs. Clinical practice guidelines emphasize the need to educate patients about the risks and benefits of treatment [6,21]. Therefore, this study created a brochure to facilitate patient education and shared decision making. It also elicited the perceptions of physicians and doulas about the brochure content and its use in clinical practice. The qualitative analysis of participant feedback yielded three overarching themes: (1) suggested uses and settings of use, (2) content revisions, and (3) perceptions about the brochure.

This study identified the possible use of the developed brochure to facilitate patient education and counseling in diverse settings, such as emergency rooms, outpatient settings, and community spaces. Clinicians in our study recommended using the brochure in discussions rather than handing out brochures without conversations. Patients and caregivers were satisfied with nurses providing brochures after verbal education in one study [22]. Patient education or conversations when providing brochures may be feasible in some of the settings suggested by participants in this study. For example, having conversations with patients in a health center or an outpatient setting may be possible. However, discussing brochures in some community spaces, such as bus or train stations, may be difficult. It is not clear if a brochure without education or conversations would be an effective method of disseminating information to patients. However, there have been previous suggestions to distribute patient educational materials in similar settings [23]. Future studies could assess patients’ preferences about modes of disseminating brochures and the efficacy of the different methods in supporting patient education and shared decision making.

This study identified several opportunities for revising the brochure’s content, including the presentation of risk information. This study highlighted the importance of presenting the risks of not receiving treatment for opioid use disorder in a neutral, non-stigmatizing, or fear-triggering message frame. Language that is judgmental or blaming should be avoided in written educational materials [24]. One of the modifications on the flyer included the word “Medication Assisted Treatment”, which has been argued to contribute to the underutilization of treatment due to potential stigma against medications for opioid use disorders [25]. Our study also highlighted the need to include affiliations in the brochure to increase the trustworthiness and credibility of the material to patients. It is documented that including the names and affiliations of the authors on written educational materials may improve the material’s credibility [24]. An additional thought on including affiliations in the brochure was for the purpose of referring to the affiliation to ask questions or seek treatment.

Based on our findings, presenting pictures or graphics that capture individuals of diverse ethnicities and races was important. Capturing diverse ethnicities relates to the cultural appropriateness of the material, which is strongly encouraged [26]. The study also found that decreasing the textual information and increasing illustrations were preferred by some individuals. However, the existing literature cautions against using illustrations when they are not essential because they could be distracting [24].

### Strengths and Limitations

This study explored the perception of providers and doulas, who serve as patient advocates. A future study should assess the acceptability, feasibility, and efficacy of using the developed brochure for patient education about treatment options. Future studies should also seek feedback on the brochure from patients. The readability of the brochure and its impact on patient knowledge or involvement in shared decision making were not assessed and should be considered by a future study. A strength of this study is the utilization of feedback from a diverse group of physicians and doulas to refine an educational brochure for pregnant women with opioid use disorders. Additionally, the draft brochure was based on published evidence obtained through a review of the literature. Future studies should explore other formats, such as interactive multimedia, to support those who may be unable to read.

## 5. Conclusions

This study provides valuable insights into the diverse settings and desired content of an educational brochure describing treatment options for pregnant women with OUDs. The brochure may be used in emergency rooms, outpatient clinics, and community spaces such as train or bus stations to educate pregnant women with OUDs. This study highlights the essential aspects of relevant education from the provider standpoint, including areas of emphasis such as the safety of medication for OUDs while breastfeeding and reduced chances of NOWS with treatment compared to no treatment. The study contributes to the literature on educational interventions to support patient engagement in treatment for OUDs. The brochure may help empower pregnant women with OUDs to engage in shared decision-making conversations with the providers and to be better involved in their care.

## Data Availability

The raw data supporting the conclusions of this article will be made available by the authors on request.

## Appendix A

- Images of pregnant women and their partners were generated via Artificial Intelligence using Youchat.

**Table A1:** Demographic information of study participants.

Characteristic	n (%)
Age, mean (SD)	35 (5.5)
Sex (Female)	16 (84.2)
Race	
Black	8 (42.1)
White	7 (36.8)
Asian	4 (21.1)
Ethnicity	
Hispanic or Latino	1 (5.3)
Non-Hispanic or Latino	18 (94.7)
Role	
Professional Doula	6 (31.6)
Resident Physician	7 (36.8)
Attending Physician or Fellow	6 (31.6)
Number of Years Spent in Caring for Pregnant Women with Opioid Use Disorders	
1–5 years	15 (78.9)
6–10 years	3 (15.8)
>10 years	1 (5.3)
Household Income	
USD 25,000–49,999	2 (10.5)
USD 50,000–74,999	8 (42.1)
USD 75,000–99,999	4 (21.1)
USD 100,000 and above	5 (26.3)

## Appendix B

**Table A2:** Themes, Subthemes, and Quotes.

Themes and Subthemes	Quotes
Suggested uses and settings of use	
(a) Settings of use	“Yeah, I think this is, um, probably really good to have in the emergency departments and maybe in the community”. P19—resident
	“I think the health centers are really great <to distribute the brochure>. A lot of them are like mainly Spanish speaking, I think it would have to be like in another language as well. But like, bus stops, SEPTA stations, um, like Planned Parenthood, Philadelphia Women’s Center, um, churches”. P19-resident
	“I think the ideal time to use this type of brochure would be like, when I see someone in the office who has substance use disorder but they like, want more information and aren’t ready to commit right away. I could provide this for them and- and discuss and say, I wanna give you some material. I want you to look it over, talk to me about it and kind of go through it and say like, I wanna give you some time to think on it”. P11—Attending
(b) Brochure uses	“Because I think especially sometimes at least when we meet them in the clinic, or when they come for any hospitalization, they’re either in withdrawal, or they’re in distress, or they’re anxious. So I think it’s always good to take something home”. P1—Attending
2. Content Revisions	
(a) Suggestions to provide additional information	“I think the other thing you could add is that buprenorphine, like after a while, can be sent home as a prescription. Like, so they don’t necessarily have to go to a clinic every day, but with methadone that you do need to be able to go to a clinic every day. Um, ‘cause a lot of people don’t know that fact”. P17—Resident
	“We can’t offer buprenorphine to people who have been previously incarcerated, or if we don’t think they’re reliable, since basically with Suboxone we give them a whole bunch and they just take it, but with methadone, we have to see them every day. So that’s why I feel bad... Because a lot of people are like, “I want Suboxone, it’s easy”, but I can’t because of their history”. P19—Resident
	“I could be wrong, Isn’t methadone for maintenance, at least in pregnant women only given us a solution? like as a liquid? I don’t think we can give any, at least not in the hospital. We only give it as a liquid”. P1—Attending
	“I think it’s always good to take something home. That is like short, that they can read. And that has like good contact information”. P1—Attending
	“methadone clinics in particular, prioritize pregnant women in terms of like, once we stabilize them in the hospital, they get priority …whereas if they were not pregnant, they kind of go to the end of the line. So I always emphasize that in addition to doing what’s like best for you and the baby, it’s also kind of the easiest time for you to establish care and kind of get plugged into an opioid use program”. P3—Attending
	“So, I would, I would definitely want to put that in there. It’s safe in breastfeeding and may help treat withdrawal in the infant. And the other thing is, the other language that I’ve used in talking about this is..it’s safe in breastfeeding in the absence of other contraindications, right? Like you’re going to have patients who have bleeding or cracked nipples or HIV”. P2—Attending
	“the APGAR... I just think this might be like too much detail. ... Um, because this is just a lot of information”. P6—Attending
	“Either say, “Overall, there’s no difference in neonatal outcomes”, or if there is a difference, then say what the difference is. And do you mean the five-minute Apgar score or the one-minute Apgar score? I would remove that <APGAR score> and talk about in general, neonatal outcomes between the two <medications>”. P6—Attending
	“What does the acronym NOWS stands for?”P4—Doula
(b) Perceptions on including detoxification as an option	“but if it <detoxification> is not recommended, I don’t know if it’s necessary to write it”. P1—Attending
	“In the options, it seems like detox should be in number three. Right? No treatment is different from detox. You know, which is a, you know, tapering down or some kind of symptom control while you, um, withdraw, etcetera. So, it should be formatted similarly. I would like start with no treatment... detox, um, which is great. You know, the bottom line with detox is it’s, it’s really great if it works. The problem is, it doesn’t work, you know, most times. but there are some people who are maybe on, a low dose or newly, addicted, that it might be an option for. And so, uh, it’s more like, you want to help the patient come to the conclusion that it’s not the best option for them. But this is kind of telling them it’s not the best option for them. I hope that makes sense”. P6—Attending
	“I really like this brochure, mostly because this is the first time, I’ve ever seen people explain why we don’t use the term detox. Um, like people come in, and they’re like, “I want a detox”. And we’re like, “That’s not exactly what’s going to be happening”. P19—Resident
(c) Wording of content	“I’m looking at the wording … the no treatment portion feels almost like fear-based um, I don’t want to say a scare tactic. But a lot of times people have issues around the wording of certain things and feel as though they’re being pressured into it”. P4—Doula
	“A lot of our patients only have like a middle school education or even less than that, so sometimes like the language just has to be brought down a little bit”. P7—Attending
(d) Pictures	“And I feel like in general, if we’re gonna have pictures of people, I would have all different races identified ‘cause a lot of our patients are from various racial backgrounds and I think people wanna see that reflected in a brochure that they might have”. P11—Attending
	“I might change the initial picture just because a lot of women who are coming are not that pregnant. So, it may be better to like, capture people with an image that’s like pretty early in pregnancy, or a cartoon picture of like a woman or something, or even something that’s not a person”. P11—Attending
	“And then I would choose a different picture of the baby. It kind of looks like its gray. Like, the baby has passed away. (laughs)” P11—Attending
Perceptions about the brochure	“Okay, I like it <brochure> a lot. I think this addresses a lot of patient concerns because patients are really worried about their babies withdrawing”. P14—Resident
	“I actually love this <brochure>. I love this because it breaks it down and you can tell them things. But like myself, I’m a visual person. You can tell me, but I like to see it. And I think that this is helpful for them actually seeing it”. P13—Doula
	“I think overall it’s really comprehensive, and thorough”. P16—Resident
